# miR-125a-5p inhibits NF-κB by negatively regulating TRAF2 to alleviate inflammatory injury in ischemic stroke rats

**DOI:** 10.1016/j.ibneur.2026.09.010

**Published:** 2026-09-21

**Authors:** Shourui Niu, Chujia Guo, Jinfen Zhang, Chunyan Li, Heng Zhu, Hongyu Liao, Jinwei Yang, Wei Ma, Liyan Li

**Affiliations:** a Institute of Neuroscience, Kunming Medical University, Kunming, Yunnan, 650500, China; b Department of Gastrointestinal and Bariatric Metabolic Surgery, First People's Hospital of Yunnan Province, Kunming, Yunnan, China; c Department of Neurology, The Second Affiliated Hospital of Kunming Medical University, Kunming, Yunnan, 650000, China

**Keywords:** Ischemic stroke, MiR-125a-5p, TRAF2, NF-κB, Inflammatory injury

## Abstract

The expression of certain miRNAs are significantly associated with both inflammatory and ischemia-reperfusion (I/R) injury in ischemic stroke. In this study, we identified cerebral cortex miR-125a-5p via RNA sequencing, which showed significant enrichment in inflammatory pathways. Cerebral infarction volume and neurological deficits were evaluated using TTC staining, mNSS scoring, and the open field test. Nissl staining and TUNEL assays were used to assess cortical apoptosis. TRAF2, NIK, and p-NFκB levels in the cerebral cortex, as well as serum TNF-α, IL-1β, and IFN-γ concentrations, were measured by Western blot and ELISA. We found that TRAF2, a predicted target of miR-125a-5p, is linked to NF-κB pathway. Overexpression of miR-125a-5p reduced infarct volume, inhibited apoptosis and neurological deficits, and downregulate TRAF2, NIK and p-NF-κB in the cortex, along with TNF-α, IL-1β, and INF-γ in the serum of I/R rats. The miR-125a-5p negatively regulates TRAF2, alleviating inflammation-induced I/R injury by modulating the NF-κB pathway.

## Introduction

The occurrence of ischemic stroke can primarily be attributed to cerebral ischemia caused by vascular embolism in the brain (Jolugbo and Ariëns, 2021)^.^ In recent years, there has been a notable increase in the incidence of disability and mortality associated with stroke (Tu et al., 2023). The pathophysiology of ischemic stroke is complex, involving neuronal cell death from ischemia and reperfusion, calcium overload, oxygen free radical production, inflammatory vesicle formation, activation of inflammatory pathways, and intricate molecular regulatory mechanisms (Zhao et al., 2022). The inflammatory response within the ischemic region of the brain triggers the activation of microglia, cerebrovascular endothelial cells and astrocytes, leading to the production of inflammatory cytokines and chemokines (Thapa et al., 2023). In ischemic stroke, affected brain cells quickly produce reactive oxygen species, pro-inflammatory cytokines such as tumour-necrosis factor α (TNF-α) and interleukin-1β (IL-1β), as well as chemokines. This process triggers the infiltration of leukocyte infiltration and neuroinflammation. Leukocytes entering the brain from the bloodstream cause local inflammation, leading to cerebral edema and the release of cytokines like tumour-necrosis factor α (TNF-α) and interleukin-1β (IL-1β). Suppressing this inflammatory response may reduce ischemia-reperfusion injury (Eren and Yilmaz, 2022). Several signalling pathways mediate inflammation, such as nuclear factor kappa-B (NF-κB) and mitogen-activated protein kinase. Studies indicate that lncRNAGm4419 contributes to hypoxia-reoxygenation injury in brain microglia by phosphorylating anti-I kappa B alpha and activating the NF-κB pathway (Wen et al., 2017). In the NF-κB signaling pathway, TNF receptor-associated factor 2 (TRAF2), TNF receptor-associated factor 3 (TRAF3), and cellular Inhibitor of apoptosis protein 1/2 are recruited to the receptor upon TNF superfamily induction (Kaszycki and Kim, 2025). This promotes NF-κB-inducing kinase (NIK) stabilization and accumulation (Taniguchi and Karin, 2018, Yu et al., 2020). As an essential kinase for the activation of the noncanonical NF-κB pathway, NIK regulates diverse processes in immunity, development, and disease. TNF activates both canonical and non-canonical NF-κB pathways via tumor necrosis factor receptor 1/2 (TNFR1/2), inducing inflammation (Sun, 2017). TRAF2, as a TNF receptor-associated factor 2, exerts inhibitory effects on necroptosis through distinct mechanisms. Specifically, it directly interacts with mixed-lineage kinase domain-like (MLKL) to prevent the binding of MLKL to receptor Interacting Protein 3, thereby suppressing TNF-α-induced and actinomycin D/Z-VAD-FMK-mediated necroptosis (Petersen et al., 2026). Studies have demonstrated that the knockout of TRAF2 in the striatum of mice results in an increased infarct size, enhanced cell death, elevated microglial activation, and upregulated expression of pro-inflammatory markers 24 h post-reperfusion (Su et al., 2017, Karl et al., 2014). These findings suggest that TRAF2 plays a crucial role in I/R injury by modulating the activation of the NF-κB signaling pathway.

In ischemic stroke, miR-155 promotes T-cell-mediated inflammation, miR-let-7i modulates thrombin activity, and miR-424 protects neurons from oxidative injury caused by ROS and ischemia (O'Connell et al., 2010, Wang et al., 2020, Mahjoubin-Tehran et al., 2021). The miR-125a gene is located on chromosome 19q13 and omprises two mature forms, miR-125a-3p and miR-125a-5p, both of which have been associated with various pathological conditions (Bhatlekar et al., 2021). The expression level of miR-125a is downregulated in a variety of human malignancies, such as lung cancer (Zhang et al., 2018), ovarian cancer (Zeng et al., 2020), and breast cancer (Shishavan et al., 2022). The upregulation of miR-125a-5p in astrocytes significantly alleviated peripheral neuropathy by targeting TRAF2 in type 2 diabetic mice (Kasimu et al., 2022). Additionally, the overexpression of miR-125a-5p not only inhibited the proliferation of hepatocellular carcinoma cells but also promoted apoptosis, thereby suppressing hepatocellular carcinoma tumorigenesis ^(^Xu et al., 2019^)^. By targeting the TRAF6/NF-κB signaling axis, miR-125a-5p exhibited therapeutic potential in alleviating peripheral neuropathy and inhibiting lipopolysaccharide (LPS)-induced acute inflammatory responses in the kidney (Yang et al., 2022). Limited studies have investigated the role of miR-125a-5p in cerebral I/R injury, and its underlying mechanisms remain unclear.

The aim of this study was to investigate the potential correlation between miR-125a-5p and TRAF2, as well as to elucidate the mechanism by which miR-125a-5p regulates the inflammatory response in ischemic stroke via the NF-κB signaling pathway.

## Materials and methods

### Animals

A total of forty-eight adult male SD (Sprague-Dawley) rats, weighing 230–260 g, were obtained from the Experimental Animal Centre of Kunming Medical University, China. All rats were SPF-grade [License No. SCXK (Dian) 2015–0002]. The rats were housed in a standardized SPF-level facility with controlled temperature, humidity, and light cycles, and were provided with ad libitum access to food and water. The rat experiments conducted in this study strictly adhered to the guidelines for the care and use of laboratory animals, and received approval [Kmmu202100001410] from the Animal Research Ethics Committee of Kunming Medical University. The surgical procedures were performed under anesthesia to minimize the discomfort experienced by the rats during the interventions. The study exclusively employed male rats to control for potential gender-related confounding variables. A total of forty-eight adult male SD rats were randomly divided into four groups: the Sham group (n = 10), the middle cerebral artery occlusion/reperfusion‌ (MCAO/R) group (n = 10), the MCAO/R + up miR-125a-5p group (n = 14), and the MCAO/R + NC group (n = 14). Rats in the MCAO/R + up miR-125a-5p group were stereotactically administered an overexpression miR-125a-5p lentiviral vector (Inject 2 ×10⁹ TU/ml lentivirus into 3 sites of rat right cortex via stereotactic coordinates, 2 μL per site. Genechem, Shanghai China) 21 days prior to MCAO/R modeling. In contrast, rats in the MCAO/R + NC group received a lentiviral negative-control injection 21 days prior to the modeling procedure.

### Establishment of a rat middle cerebral artery embolism/reperfusion (MCAO/R) model

The rats were anesthetized via intraperitoneal injection of 2% pentobarbital (30 mg/kg, milliporeSigma). Subsequently, the carotid artery was exposed and the right common carotid artery (CCA), internal carotid artery (ICA), and external carotid artery (ECA) were carefully dissected. The ECA and CCA were ligated using silk thread, while the ICA was temporarily occluded with a microvascular clamp. A small incision was made in the CCA, and nylon monofilament sutures were inserted into the ICA and advanced distally to occlude the origin of the middle cerebral artery (MCA). In the sham group, the same procedure was performed except that the suture was not inserted into the ICA. After 2 h of occlusion, the nylon suture was withdrawn to restore reperfusion. The body temperature of the rats was maintained at 37.0 ± 0.5 ℃ throughout the surgical procedure using a heating pad. The cerebral blood flow in the MCAO/R rats was continuously monitored with a laser speckle Doppler flowmeter (RFLSI III, RWD Life Science). Rats with a blood flow reduction of less than 30% and/or an mNSS score lower than 7 points were excluded from the study.

### miRNA sequencing and KEGG functional enrichment analysis

The total RNA for RNA sequencing was extracted from the brain tissues of infarct areas using TRIzol reagent (Life Technologies, Carlsbad, CA), with three samples randomly selected from each group (n = 3 in each group). The purity of RNA was evaluated by measuring the OD260/OD280 ratio with a NanoDrop spectrophotometer. The concentration of RNA was quantified using the Qubit RNA Assay Kit (Thermo Fisher Scientific) for precise quantitative analysis. The qualified samples were subsequently used for library construction with the Small RNA Sample Prep Kit. RNA integrity was evaluated using the Agilent 2100 Bioanalyzer system. The library was initially quantified using the Qubit 2.0 Fluorometer and diluted to a concentration of 1 ng/µl. The effective concentration of the library was precisely determined by quantitative real-time polymerase chain reaction (qPCR) to ensure high-quality sequencing. Subsequently, the library was subjected to high-throughput sequencing for further analysis. For the analysis of the Kyoto Encyclopedia of Genes and Genomes (KEGG) (Kanehisa et al., 2017), we utilized the KOBAS software to statistically evaluate the enrichment of differentially expressed genes and miRNA target genes within KEGG pathways ^(^Bu et al., 2021^)^.

### Reverse transcription-quantitative polymerase chain reaction (RT-qPCR)

The miRNA was extracted from cells and cerebral tissue using the miRcute miRNA Extraction Kit (Tiangen Biochemistry, Beijing). cDNA synthesis was performed with the miRcute Enhanced miRNA cDNA Synthesis Kit at 42°C for 30 min, followed by denaturation at 95°C for 3 min. RT-qPCR analysis was conducted using the miRcute Enhanced miRNA Fluorescence Quantitative Detection Kit (Tiangen Biochemistry, Beijing). Total RNA extraction was done using TRI Reagent (Invitrogen, Carlsbad, CA, USA), and cDNA was synthesized using the PrimeScript™ IV 1st Strand cDNA Synthesis Mix (Takara, Beijing, China). Real-time PCR was performed with the KAPA SYBR FAST qPCR Kit Master Mix, following a protocol of enzyme activation at 50°C for 2 min, initial denaturation at 95°C for 10 min, and 40 cycles of denaturation at 95°C for 15 s and annealing at 60°C for 1 min. Primer sequences are listed in supplemental table 1.

### Cell culture and oxygen glucose deprivation/reperfusion (OGD/R) model

The BV2 cells (Wuhan Pricella Biotechnology Co., Ltd, Wuhan, China) were cultured in DMEM medium supplemented with 10% fetal bovine serum, 100 IU/ml penicillin and 100 μg/ml streptomycin. Cells were incubated at 37℃ in a 5% CO_2_ humidified chamber, with medium changes every 2–3 days. BV2 cells in the logarithmic growth phase under normal culture conditions were divided into the control group and the OGD/R group. The control group cells were cultured in standard DMEM medium with normal glucose and oxygen levels. The OGD/R group cells were cultured in glucose-free DMEM medium under hypoxia conditions (5% CO2 and 95% N2) at 37℃ for 2 h.

### Nissl staining, Hematoxylin and Eosin (HE) staining, and Terminal deoxynucleotidyl transferase dUTP nick end labeling (TUNEL) staining

The rats were transcardially perfused with normal saline and 4% paraformaldehyde (Sigma) 24 h after MCAO. Brains were removed, post-fixed in 4% paraformaldehyde for 24 h, and embedded in paraffin. Four-micrometer sections were cut, dewaxed in xylene for 45 min, and dehydrated with gradient ethanol. Sections were stained with Nissl reagent (ServiceBio) and hematoxylin and eosin (HE) according to the manufacturer's instructions (ServiceBio). Apoptotic cells in the rat cerebral cortex were identified using the TUNEL assay with an apoptosis detection kit (ServiceBio).

### 2,3,5-triphenyltetrazolium chloride (TTC) staining

After 24 h of reperfusion, rats in each group (n = 3) were anesthetized with 2% pentobarbital (30 mg/kg), decapitated, and their brains rapidly removed for TTC staining. Brains were sectioned into six 2-mm-thick coronal slices, stained with 2% TTC solution for 30 min at 37°C, and then fixed overnight in 4% paraformaldehyde. Finally, the slices were transferred to saline. The computer imaging analysis system captured six images per group. ImageJ software (https://imagej.nih.gov/ij/) was used to measure cerebral infarction volume, calculated using the formulae described by Lin et al (Lin et al., 1993).

### Evaluaton of neurological function

The neurological deficits in rats were evaluated using the modified neurological severity score (mNSS) at 1, 3, and 7 days post-reperfusion. This scale includes assessments of motor function, sensory perception, balance control, and reflex responses. Scores range from 0, indicating minimal impairment, to 18, representing maximal deficits, with higher scores reflecting more pronounced neurological abnormalities (Garcia et al., 1995).

### Open field experiments

The open field test was utilized to assess the spontaneous locomotor activity of the rats at 1, 3, and 7 days post-reperfusion. A detailed description of the methodology is provided in a prior study (Kraeuter et al., 2019). The experimental data were collected and analyzed using the Activity Monitor animal behavior analysis system.

### Enzyme-linked immunosorbent assay (ELISA)

Serum samples were collected from each group of rats, and specific ELISA kits (Jianglai Bio, Shanghai, China) were employed to quantify the serum levels of TNF-α, IL-1β, caspase-8, and IFN-γ. Absorbance values were measured at 450 nm using a microplate reader.

### Western blot analysis

Protein extracts from rat cerebral tissues were isolated using RIPA buffer. Protein concentrations were measured with a BCA assay kit (P0010, Beyotime). Equal protein samples (25 µg) were separated by 12% SDS-PAGE and transferred to PVDF membranes. The membranes were blocked with 5% skim milk for 1 h at room temperature, incubated overnight at 4 ℃ with primary antibodies (Supplemental Table 2), and then incubated with a goat anti-rabbit IgG antibody (1:5000, Bioss) for 2 h. Membranes were visualized using enhanced chemiluminescence and analyzed with ImageJ software.

### Statistical analysis

The data were analyzed using GraphPad Prism 8.0 and expressed as mean ± SEM, showing a normal distribution. An independent samples *t*-test (two-tailed) was used to compare two groups, with p < 0.05 indicating significance. For multigroup comparisons, one-way ANOVA followed by Bartlett's correction (GraphPad Prism 7.0) was applied.

## Results

### Establishment of rat MCAO/R model

The cerebral blood flow were continuously monitored using a laser scatter imaging system, both before and 24 h following the establishment of the MCAO/R model. The rats in the MCAO/R group exhibited a significant decrease in cerebral blood flow compared to those in the Sham group (Supplemental figure 1).

### Enrichment analysis of differentially expressed miRNAs in rats with ischemic stroke

The application of hierarchical clustering revealed consistent variations in miRNA expression profiles among different samples within the same group in ischemic stroke rats (Fig. 1A). A total of 264 differentially expressed miRNAs were identified in the IR group compared to the Sham group, with 137 miRNAs showing up-regulation and 127 miRNAs exhibiting down-regulation (Fig. 1B). The KEGG enrichment analysis of the differentially expressed miRNAs revealed significant enrichment of target genes in inflammatory injury-related signaling pathways and chemokines, such as NF-κB, Wnt, VEGF, ErbB, apoptosis, fatty acid elongation, lysosome, focal adhesion, and others (Fig. 1C). GO term analysis revealed the functional roles of the target genes associated with six miRNAs related to inflammation. Based on the KGEE and GO enrichment analysis results, we selected the NF-κB signaling pathway for further study, as it closely associated with inflammatory injury following I/R injury.

**Fig. 1:**
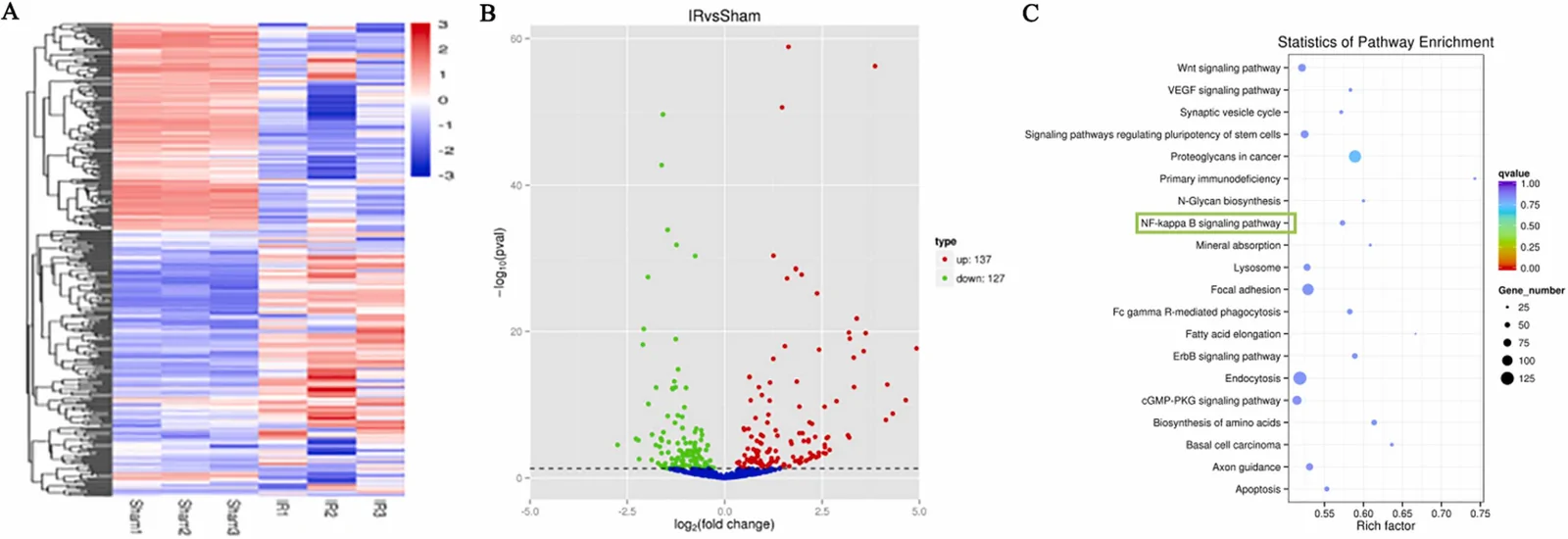
The analysis of differential expression miRNAs in rats with ischemic stroke. A: Hierarchical cluster analysis of differentially expressed miRNAs. B: Volcano plots of differentially expressed miRNAs. C: KEGG enrichment analysis of differential expression miRNAs.

### Validation of candidate miRNAs by qRT-PCR

We identified six miRNAs (miR-125a-5p, miR-212–3p, miR-351–5p, miR-504, miR-3085, and miR-133c) that were significantly enriched in NF-κB signaling pathway when comparing the MCAO/R group to the Sham group. The expression levels of miR-3085 and miR-351–5p were upregulated in the IR group, whereas the expression levels of miR-212–3p and miR-133c were downregulated in the MCAO/R group compared to the Sham group, which was consistent with the sequencing results (Fig. 2A-F). The predicted target gene of miR-125a-5p was identified as TRAF2 (Supplemental figure 2), which is associated with the NF-κB signaling pathway, using the TargetScan database. TRAF2 is enriched in the NF-κB signaling pathway and significantly upregulated in brain tissue of the IR group and BV2 cells of the OGD/R group compared to the sham and normal groups, as shown by qRT-PCR (Fig. 2G，Fig. 2I). The expression levels of miR-125a-5p were significantly downregulated in the brain tissue of the IR group and BV2 cells of the OGD/R group compared to the normal and sham groups, as shown by qRT-PCR analysis (Fig. 2H).

**Fig. 2:**
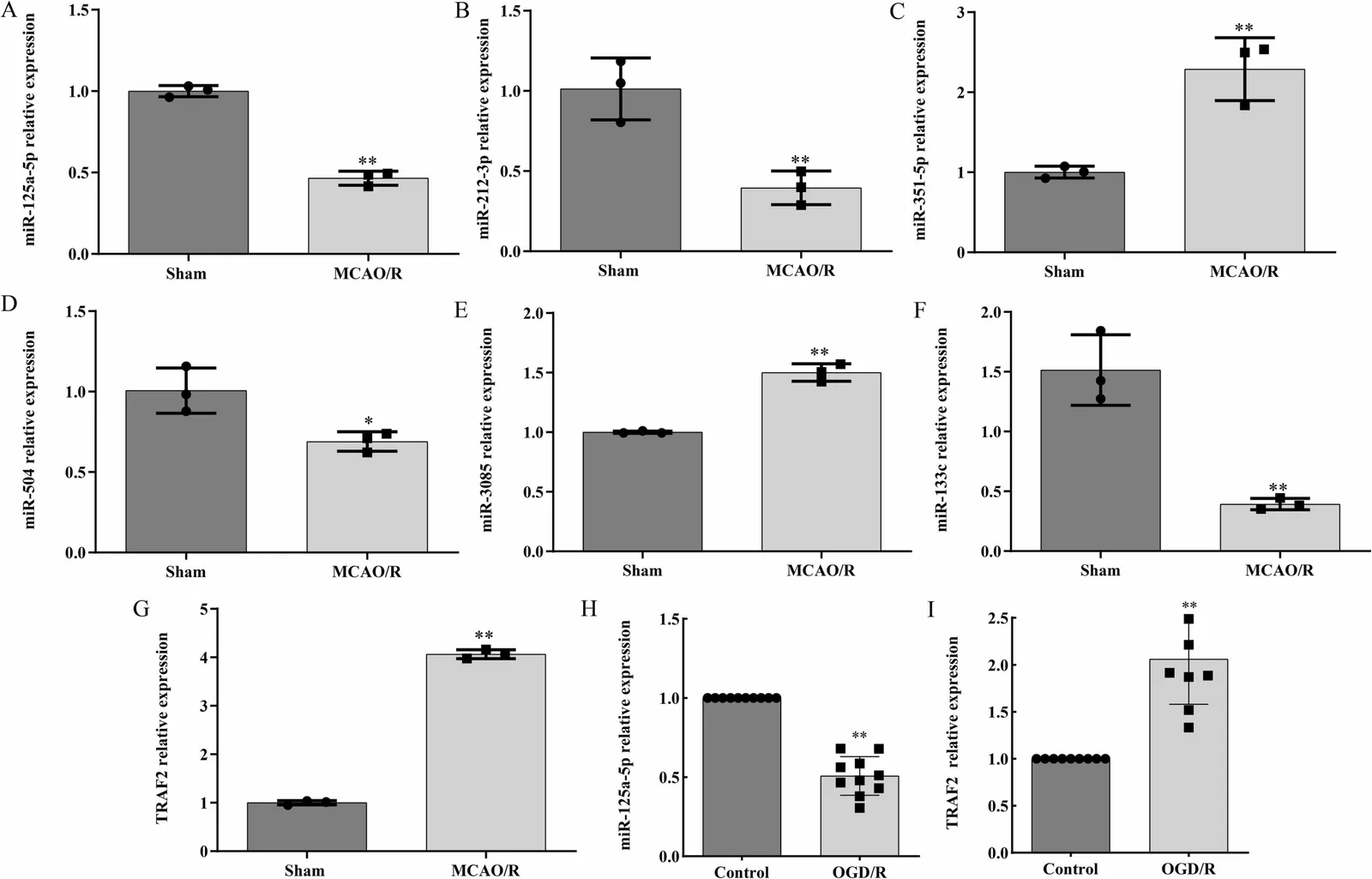
The miRNA expression levels and target gene prediction of miR-125a-5p in rat brain tissue with ischemic stroke were validated. A-F: Validation of miRNA expression levels in rat brain tissue by qRT-PCR. The expression levels of miR-125a-5p, miR-212–3p, miR-351–5p, miR-504, miR-3085 and miR-133c were quantified in the Sham and MCAO/R groups. G: The expression levels of TRAF2 in the brain tissues of rats with ishcemic stroke. H: miR-125a-5p expression in BV2 cells. I: TRAF2 expression in BV2 cells. *p < 0.05, ^**^p < 0.01.

### Overexpression miR-125a-5p alleviate I/R injury in ischemic stroke rats

The expression of miR-125a-5p was significantly increased in the overexpression miR-125a-5p group compared to the control group, confirming successful overexpression (Supplemental figure 3). TTC staining showed a significant infarcted area in MCAO/R rat brains compared to Sham rats. The infarcted area was reduced in MCAO/R + up miR-125a-5p rats compared to MCAO/R rats (Fig. 3A-B). Compared with the Sham group, the MCAO/R group had significantly fewer apoptotic cells and Nissl staining-positive cells in the cerebral cortex (Fig. 3C-G). In contrast, compared with the MCAO/R group, the MCAO/R + up miR-125a-5p group exhibited reduced apoptosis and a higher number of Nissl staining-positive cells (Fig. 3C-G).

**Fig. 3:**
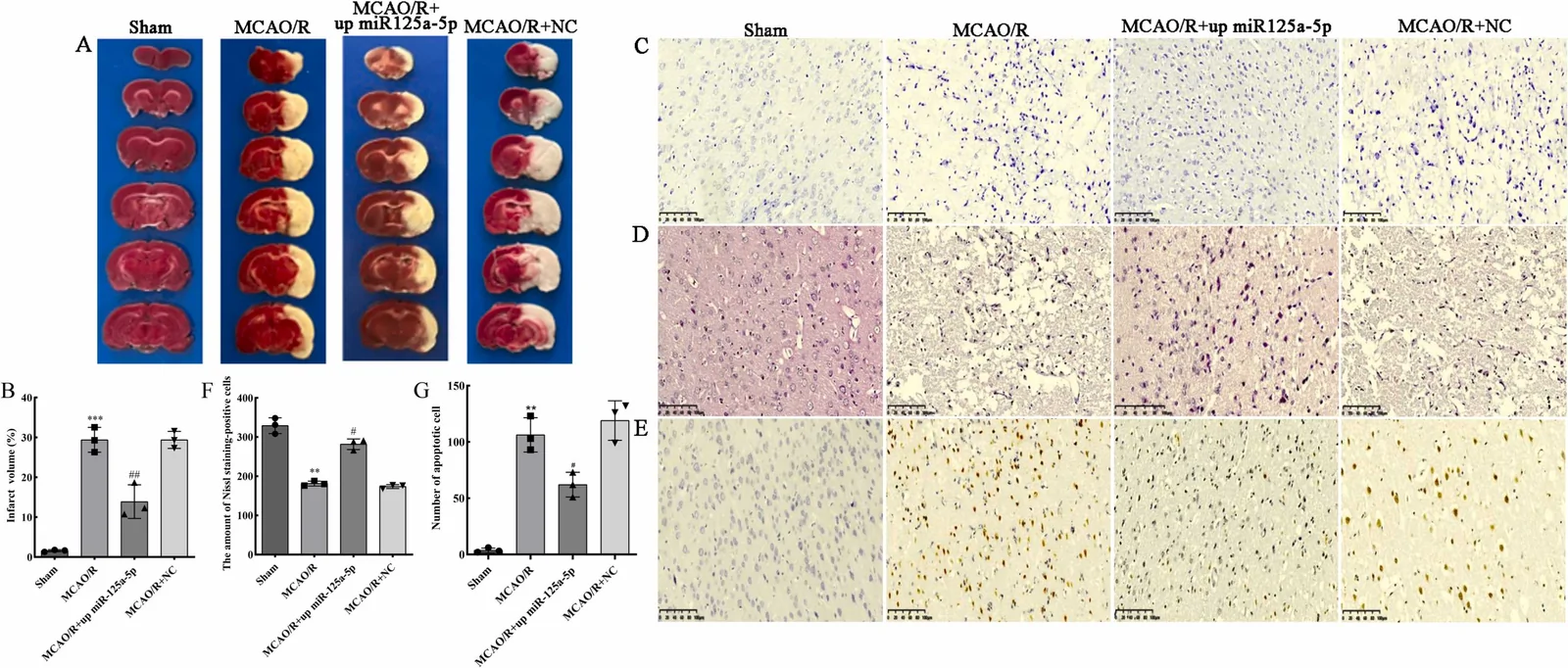
The extent of cerebral infarction and apoptosis was detected in rats with ischemic stroke following the overexpression of.miR-125a-5p. The rats were divided into four groups: Sham group, MCAO/R group, MCAO/R + up miR-125a-5p group, and MCAO/R + NC group. A: TTC staining showing the area of cerebral infarction in each group. B: the volume of infarction were quantitatively analyzed in each group of rats. C-E: The representative images of Nissl's, HE and TUNEL staining in cerebral cortex tissue in each group, respectively. The magnification is 100x. Data are expressed as mean±SD. ^***^p < 0.001 compared with sham group, ^##^p < 0.01 compared with MCAO/R group.

### Overexpression of miR-125a-5p enhances locomotor and neurological recovery in rats following ischemic stroke

The MCAO/R group exhibited a significant decrease in ambulatory distance and an increase in resting time, as well as higher mNSS scores compared to the Sham group (Fig. 4A-D). However, rats in the MCAO/R + up miR-125a-5p group exhibited significantly increased ambulatory distance, decreased resting time, and lower mNSS scores compared to those in the MCAO/R group on days 1, 3, and 7 following ischemia-reperfusion (Fig. 4A-D).

**Fig. 4:**
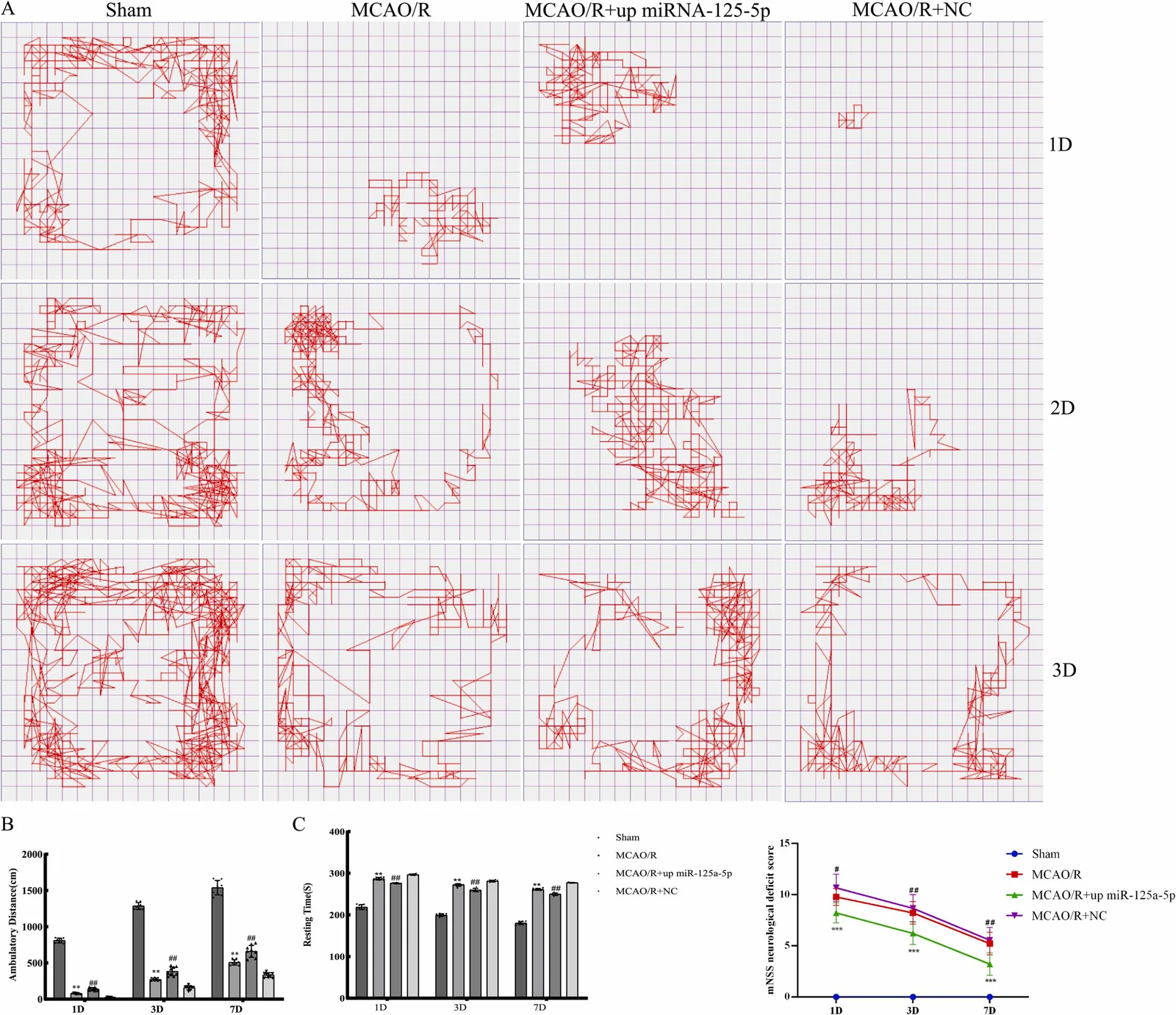
Behavioral scores of neurological impairment and open field in ischemic stroke rats following ischemia-reperfusion at 1, 3 and 7 days. A: The representative images of locomotor trajectories in the open field of rats for each group. B-C: The ambulatory distance and resting time in the open field were quantitatively assessed for each experimental group of rats. D: Modified neurological severity scores of rats in each group. Data are expressed as mean±SD. ^***^p < 0.001 compared with sham group. ^#^*p* < 0.05, ^##^*p* < 0.01 compared with MCAO/R group.

### Overexpression of miR-125a-5p inhibits inflammatory factor expression in ischemic stroke rats

The expression of miR-125a-5p was significantly decreased in the MCAO/R group compared to the Sham group at 1, 3, and 7 days. In contrast, the MCAO/R + up miR-125a-5p group showed increased expression levels at 1, 3, and 7 days compared to the MCAO/R group (Fig. 5A-C). The serum levels of TNF-α, IL-1β, Interferon-γ (INF-γ), and Caspase-8 were significantly increased in the MCAO/R group at 1, 3, and 7 days compared to the Sham group. In the MCAO/R + up miR-125a-5p group, significantly the serum levels of TNF-α, IL-1β, INF-γ, and Caspase-8 were significantly decreased compared to the MCAO/R group (Fig. 5D-G).

**Fig. 5:**
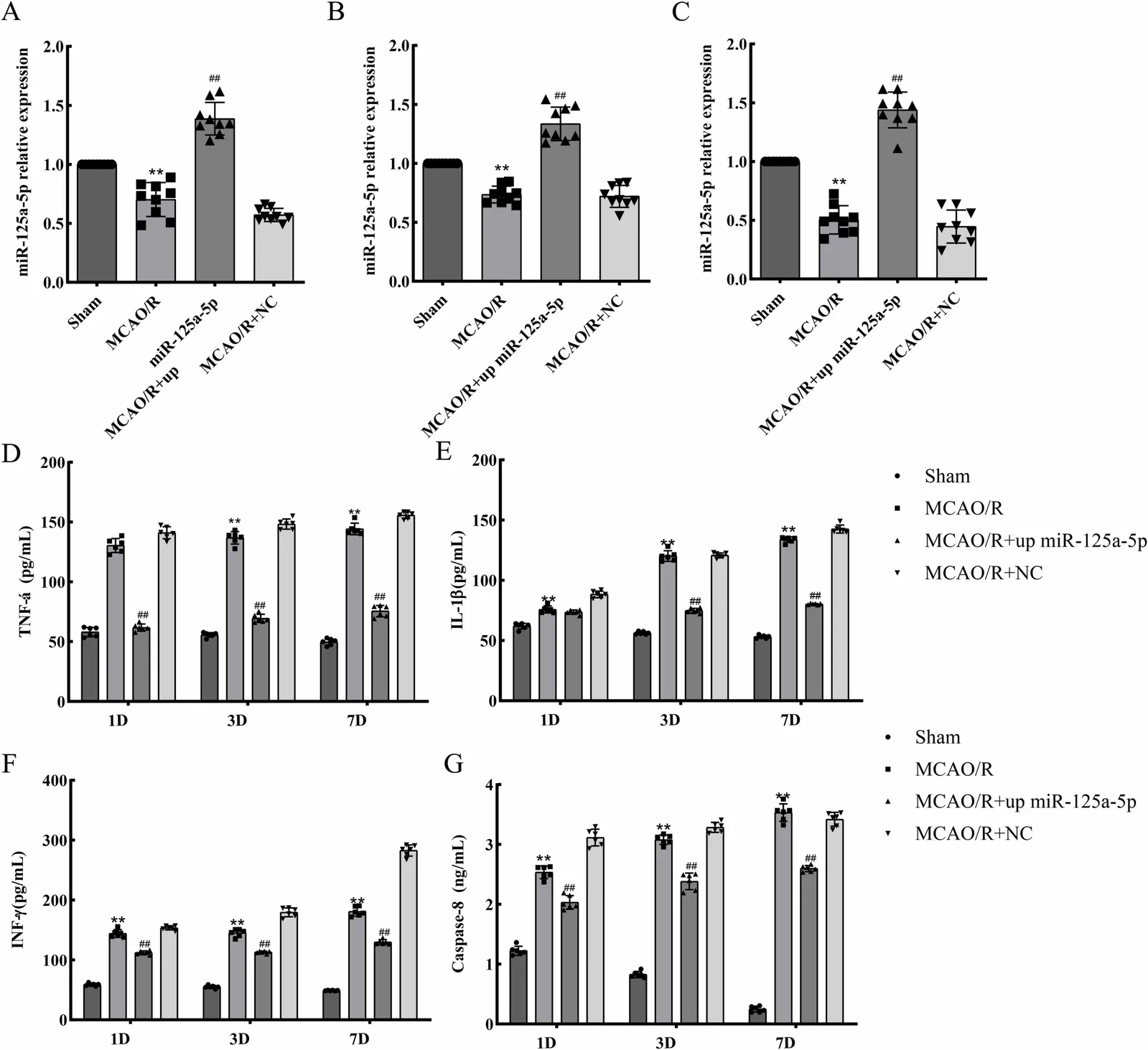
The expression of miR-125a-5p and inflammatory factors in serum and cerebral cortex tissue following ischemia-reperfusion at 1, 3 and 7 days. A-C: The expression of miR-125a-5p in rat cerebral cortex tissue following ischemia-reperfusion at 1, 3 and 7 days were detected by qRT-PCR. D-G: The levels of TNF-α, IL-1β, INF-γ and Caspase-8 in rat serum following ischemia-reperfusion at 1, 3 and 7 days were quantified by ELISA. Data are expressed as mean±SD. * *p* < 0.05, ^**^p < 0.01 com*p*ared with sham group. ^#^*p* < 0.05, ^##^*p* < 0.01 compared with MCAO/R grou*p*.

### The miR-125a-5p negatively regulates the NF-κB signaling pathway in ischemic stroke rats

The expression levels of TRAF2, NIK, and p-NF-κB proteins were significantly increased in the cerebral tissues of rats in the MCAO/R group after 3 days of ischemia-reperfusion compared to those in the Sham group (Fig. 6A-D). In contrast, the expression of TRAF2, NIK, and p-NF-κB proteins was significantly decreased in the cerebral tissues of rats in the MCAO/R + up miR-125a-5p group when compared to the MCAO/R group (Fig. 6A-D). This indicates that miR-125a-5p negatively regulates the NF-κB signalling pathway by modulating TRAF2 expression.

**Fig. 6:**
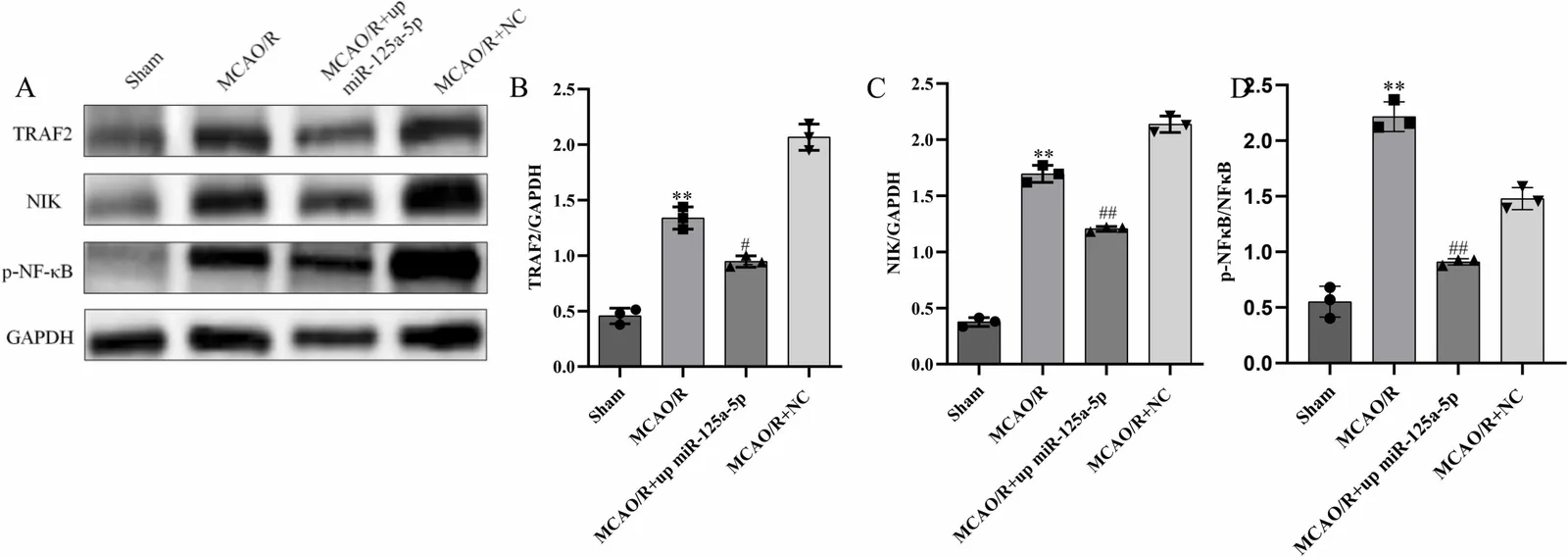
The protein levels of TRAF2, NIK and p-NF-κB were assessed in ischemic stroke rats with miR-125a-5p overexpression. A: the representative protein bands. B-D: the protein expression of TRAF2, NIK and p-NF-κB in the cerebral cortex after overexpression of miR-125a-5p in ischemic stroke rats. ^**^p < 0.01 compared with sham group, ^#^*p* < 0.05, ^##^*p* < 0.01 compared with MCAO/R group.

## Discussion

The process of I/R is associated with an increase in oxidative stress and the release of pro-inflammatory factors from cells, which subsequently trigger a series of pathological cascades, including apoptosis, disruption of the blood-brain barrier, cerebral edema, and hemorrhagic transformation, either directly or indirectly (De Meyer et al., 2022, Jurcau and Ardelean, 2021, Lu et al., 2026). The neuroinflammatory mechanisms, which are closely associated with oxidative stress, play a potencial role in inducing neuronal damage during the acute phase of cerebral ischemia. This ultimately exacerbates I/R injury and neurological deficits that resulting from the reperfusion process (Wang et al., 2021, Wang et al., 2022). The inflammatory response has three phases: the acute phase, where phagocytes and leukocytes remove necrotic cells within hours; the subacute phase, marked by inflammation resolution; and the late phase, involving tissue repair by microglia and astrocytes (Jurcau and Simion, 2021, Drieu et al., 2018). I/R injury following ischemia can induce neuronal injury. Subsequent neuronal necrosis and disruption of neuronal-microglial interactions may amplify inflammatory signaling, thereby exacerbating the extent of I/R (Liu et al., 2019). Inhibiting necroptosis significantly decreases the volume of cerebral infarction in rats with ischemic stroke (Zhang et al., 2017, Huo et al., 2024, Zhang et al., 2022). The irreversibility of neuronal damage within the core infarct zone is widely recognized. The brain region recruited by the ischemic core in the subacute phase of ischemic stroke may represent a secondary inflammatory area, which could potentially be rescued by blocking lymphocyte infiltration into the brain, thus targeting the inflammatory penumbra (Levard et al., 2021, Nong et al., 2022). The inflammatory penumbra involves T-cell infiltration into the brain's perilesional area, mediated by vascular-cell adhesion molecule-1. These cells contribute to delayed neuronal death. Preclinical data supports the use of anti-inflammatory strategies to salvage this region in acute stroke (Qiao et al., 2023). Studies show that I/R injury releases neuroinflammatory markers like TNF-α, IL-1β, and INF-γ (Li et al., 2023, Ermine et al., 2021). Additionally, increased Caspase-8 expression promotes neuronal apoptosis in brain tissue of ischemic stroke rats (Ma et al., 2020). In particular, peptides have been widely recognized as a treasure trove for drug discovery (Peng et al., 2026, Liu et al., 2026, Wang et al., 2026, Deng et al., 2026, Kang et al., 2025, Huang et al., 2025, Fu et al., 2026a, Fu et al., 2026b), and some peptides or peptide-based interventions have shown significant potency for ischemic stroke (Liu et al., 2023, Yin et al., 2022).

Some miRNAs have the capability to either activate or inhibit inflammatory signals. For example, miR-155 has been associated with various neuroinflammatory diseases (Zingale et al., 2021). Overexpression of miR-34c-5p has been demonstrated to attenuate inflammatory and apoptosis, thereby alleviating I/R injury (Tu and Hu, 2021). Conversely, inhibition of miR-3a-19p can induce IGFBP3 activation, which exerts neuroprotective effects by suppressing inflammation and apoptosis (Chai et al., 2020). The exosome BBR-exos is capable of delivering miR-182–5p to injured neurons, thereby suppressing the expression of Rac1 and subsequently inhibiting neuroinflammation in the context of ischemic stroke (Ding et al., 2023). The suppression of inflammation can be achieved by inhibiting miR-19a/b-3p through direct targeting of silent mating type information regulation 2 homolog-1 (SIRT1) mRNA. Knockdown of SIRT1 prevents FoxO3 binding to the sphingosine kinase 1 (SPHK1) promoter, thereby activating its transcription and effectively suppressing inflammation (Zhou et al., 2021).

In this study, we identified two significantly upregulated miRNAs (miR-351–5p and miR-3085) and four significantly downregulated miRNAs (miR-125a-5p, miR-212–3p, miR-504, and miR-133c) in the brain tissues of rats in the MCAO/R group compared to those in the sham group. Among these miRNAs, KEGG enrichment analysis revealed that the potential target genes of miR-125a-5p were enriched in inflammatory pathways. Furthermore, TargetScan predicted TRAF2 as a target gene of miR-125a-5p, which plays a critical role in the NF-κB signaling pathway.

We observed that the overexpression of miR-125a-5p resulted in a significant reduction in TRAF2 protein expression within the brain tissue of ischemic stroke rats. Additionally, it resulted in decreased serum levels of inflammation-related factors, including TNF-α, IL-1β, INF-γ, and Caspase-8 in ischemic stroke rats. TRAF2, a member of the TRAF protein family, is essential for TNF-alpha-mediated activation of NF-κB and serves as a mediator of the anti-apoptotic signals from TNF receptors (Mukherjee et al., 2024, Dostert et al., 2019). These findings suggest that miR-125a-5p may negatively regulates TRAF2, thereby attenuating cerebral inflammatory injury in ischemic stroke rats. A study demonstrated that miR-125a-5p inhibits the invasive capacity of hepatocellular carcinoma cells by targeting TRAF2 ^(^Wu et al., 2022^)^, while suppression of miR-125a-5p in rats attenuates cerebral ischemia-induced reperfusion injury through its targeting of IGFBP3 ^(^Song et al., 2021^)^. Electroacupuncture treatment has been demonstrated to enhance motor function and reduce neuroinflammation through the inhibition of the miR-223/NOD-like receptor protein 3 (NLRP3) pathway, thereby exerting a neuroprotective effect in rat MCAO (Sha et al., 2019). MiR-873a-5p mitigated microglia-mediated neuroinflammation and ameliorated neurological deficits in individuals with traumatic brain injury through inhibition of the NF-κB signaling pathway (Long et al., 2020). In our study, the upregulation of miR-125a-5p was found to significantly decrease the secretion of inflammation-related factors, such as TNF-α, IL-1β, IFN-γ, and Caspase-8, in the serum of rats. Moreover, miR-125a-5p facilitated the improvement of neurological function by inhibiting TRAF2, thereby suppressing the activation of the NF-κB signaling pathway.

## Conclusion

The upregulation of miRNA-125 may potentially exert a negative regulatory effect on TRAF2 to attenuate inflammatory and I/R injury via the NF-κB signaling pathway in rats with ischemic stroke.

## Abbreviation

I/R: ischemia-reperfusion
TNF-α: tumour-necrosis factor α
IL-1β: interleukin-1β
NF-κB: nuclear factor kappa-B
TRAF2: TNF receptor-associated factor 2
TRAF3: TNF receptor-associated factor 3
NIK: NF-κB-inducing kinase
TNFR1/2: tumor necrosis factor receptor 1/2
MLKL: mixed-lineage kinase domain-like
LPS: Lipopolysaccharide
SD: Sprague-Dawley
MCAO/R: middle cerebral artery occlusion/reperfusion
CCA: common carotid artery
ICA: internal carotid artery
ECA: external carotid artery
MCA: middle cerebral artery
qPCR: quantitative real-time polymerase chain reaction
KEGG: Kyoto Encyclopedia of Genes and Genomes
RT-qPCR: Reverse transcription-quantitative polymerase chain reaction
OGD/R: oxygen glucose deprivation/reperfusion
HE: Hematoxylin and Eosin
TUNEL: Terminal deoxynucleotidyl transferase dUTP nick end labeling
TTC: 2,3,5-triphenyltetrazolium chloride
mNSS: modified neurological severity score
ELISA: Enzyme-linked immunosorbent assay
NLRP3: NOD-like receptor protein 3
SPHK1: sphingosine kinase 1
SIRT1: silent mating type information regulation 2 homolog-1
INF-γ: Interferon-γ

## CRediT authorship contribution statement

**Shourui Niu:** Validation, Methodology, Data curation. **Chujia Guo:** Validation, Formal analysis, Data curation. **Jinfen Zhang:** Validation, Formal analysis, Data curation. **Chunyan Li:** Methodology, Data curation. **Heng Zhu:** Validation. **Hongyu Liao:** Formal analysis. **Jinwei Yang:** Visualization, Funding acquisition. **Wei Ma:** Writing – original draft, Methodology, Funding acquisition, Data curation. **Liyan Li:** Funding acquisition, Conceptualization.

## Ethics declaration

This study was conducted in accordance with the ARRIVE (Animal Research: Reporting of In Vivo Experiments) guidelines. This study was approved by the Animal Research Ethics Committee of Kunming Medical University. (Approval No. Kmmu202100001410)

## Funding

This work was supported by the Yunnan Fundamental Research Projects, China (grant numbers: 202501AY070001–144), the Yunnan Provincial Key clinical Specialty (Gastrointestinal Surgery) and the Yunnan Xingdian Talent Support Plan medical and health personnel project (YLWS-2023–0068), and Kunming Medical University Undergraduate Innovation Training Grant for 2025, China (202510678001).

## Declaration of Competing Interest

No potential conflict of interest was reported by the author(s).

## Data Availability

The data presented in this study are available on request from the corresponding author.

## Footnotes

Appendix A Supplementary data associated with this article can be found in the online version at doi:10.1016/j.ibneur.2026.09.010.
