# Supplementary material for: miR-125a-5p inhibits NF-κB by negatively regulating TRAF2 to alleviate inflammatory injury in ischemic stroke rats
Source: IBRO Neurosci Rep. 2026 Sep 21;21:858–67. doi: 10.1016/j.ibneur.2026.09.010 (PMC13634409; doi:10.1016/j.ibneur.2026.09.010)
Supplement: Supplementary file 1 [file mmc1.docx]

**Supplemental tab**le 1 | Primer information

| Gene name | **Sequence（5’－3’）** |
| --- | --- |
| U6 | F：TGCTTCGGCAGCACATATAC |
| miR-125a-5p | F：TCCCTGAGACCCTTTAACCTGT |
| miR-212-3p | F：TAACAGTCTCCAGTCACGGCC |
| miR-351-5p | F：TCCCTGAGGAGCCCTTTGAG |
| miR-504 | F：AGACCCTGGTCTGCACTCTGTC |
| miR-3085 | F：TCTGGCTGCTATGGCCCC |
| miR-133c | F：CAGCTGGTTGAAGGGGACC |
| GAPDH | F：CATTCTTCCACCTTTGAT |
|  | R：CTGTAGCCATATTCATTGT |
| TRAF2 | F：GCGAGAAGAAGCACCACACTGAG |
|  | R：GCCACAGCCATCACAGGTTAAGG |
