# Supplementary material for: miR-125a-5p inhibits NF-κB by negatively regulating TRAF2 to alleviate inflammatory injury in ischemic stroke rats
Source: IBRO Neurosci Rep. 2026 Sep 21;21:858–67. doi: 10.1016/j.ibneur.2026.09.010 (PMC13634409; doi:10.1016/j.ibneur.2026.09.010)
Supplement: Supplementary file 2 [file mmc2.docx]

**Supplemental table 2 Primary antibodies information**

| Primary antibody | Species | Dilution | Company |
| --- | --- | --- | --- |
| GAPDH | rabbit | 1:1000 | Affinity Biosciences |
| TRAE2 | rabbit | 1:1000 | Affinity Biosciences |
| NIK | rabbit | 1:1000 | Affinity Biosciences |
| p-NFκB | rabbit | 1:1000 | Affinity Biosciences |
